# Thymoma-Induced Autoimmune Hepatitis: A Rare Paraneoplastic Syndrome

**DOI:** 10.7759/cureus.5637

**Published:** 2019-09-12

**Authors:** Kendra T Stilwell, Sierra R Musick, Katherine M Cebe, Amilcar L Morales-Cardona

**Affiliations:** 1 Internal Medicine, Brooke Army Medical Center, San Antonio, USA; 2 Pathology, Brooke Army Medical Center, San Antonio, USA; 3 Gastroenterology and Hepatology, Brooke Army Medical Center, San Antonio, USA

**Keywords:** autoimmune hepatitis, thymoma, paraneoplastic syndrome, autoimmunity

## Abstract

Thymomas are rare neoplasms of the thymus and are often associated with immune-mediated paraneoplastic syndromes, most commonly, myasthenia gravis. The same underlying mechanism can produce antibodies to other self-antigens in various organ systems. Autoimmune hepatitis is a rare complication of thymoma. We present a 35-year-old healthy male, initially thought to have drug-induced liver injury, who was subsequently diagnosed with thymoma-induced autoimmune hepatitis, a rare syndrome of which only two previous cases have been reported.

## Introduction

Thymomas are rare neoplasms of the thymus, a primary lymphoid organ whose main function is T-cell maturation and differentiation. When genetic mutations allow this process to go unchecked, these neoplastic cells can produce antibodies to any host cell. This leads to a loss of tolerance for self-antigens, and the development of autoimmune syndromes [1]. These paraneoplastic syndromes often manifest concurrently with thymoma diagnosis, but in some cases are clinically silent until much later, sometimes even after thymoma resection owing to circulating mature T-lymphocytes that have developed autoimmunity [2]. Myasthenia gravis is most commonly associated with thymoma-induced paraneoplastic syndromes. However it is important to search for other autoimmune syndromes which can also be less commonly associated with a thymoma. 

## Case presentation

**Figure 1:**
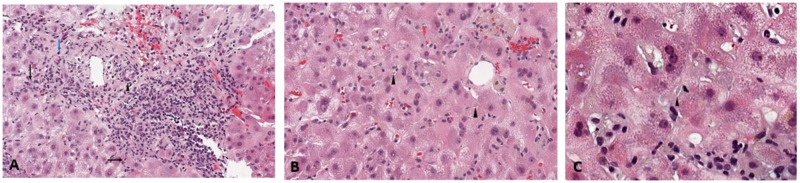
Initial Liver Biopsy - 1A: Predominant lymphocytic interface hepatitis (black arrow), with few plasma cells (blue arrow) and bile duct injury (arrowhead). 1B, 1C: Canalicular bile plugs, consistent with acute cholestasis

A 35-year-old healthy African American male was referred to the hepatology clinic for an incidental finding of asymptomatic elevated liver associated enzymes (LAEs) discovered on routine lab work by his primary care provider. On serologic evaluation, his aspartate transaminase (AST) was 995 IU/L, alanine transaminase (ALT) 1155 IU/L and total bilirubin was 14.1 mg/dL (direct bilirubin 9.4 mg/dL). A complete workup was significant for a weakly positive anti-nuclear antibody (ANA) of 1:40 speckled-pattern, anti-smooth muscle antibody (ASMA) of 1:40, and elevated immunoglobulin G (IgG) of 1663 mg/dL. Liver biopsy was performed given his weakly positive antibodies and persistently elevated transaminases after discontinuation of the workout supplement. The liver histology (Figure 1A, 1B, 1C) was non-conclusive, demonstrating predominant lymphocytic interface hepatitis with a few plasma cells. Bile duct injury was present with canalicular bile plugs, compatible with acute cholestasis. These findings are consistent with either drug-induced liver injury (DILI) or autoimmune hepatitis (AIH). DILI was favored at the time due to the presence of acute intrahepatic cholestasis with necro-inflammatory pattern of hepatic injury in the setting of long term workout supplement use.

**Figure 2:**
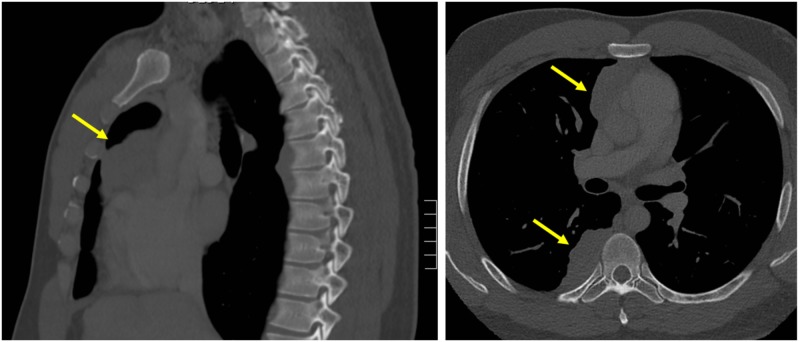
CT Chest demonstrating thymoma in anterior mediastinal space with pleural metastasis

Six months after the initial presentation to the hepatology clinic, the patient presented to his primary care provider for persistent upper respiratory symptoms and mild shortness of breath. A chest x-ray was ordered, which incidentally discovered bilateral pleural effusions and an anterior mediastinal mass. The patient underwent CT of the chest (Figure 2A, 2B) and was diagnosed with a thymoma with metastasis to the pleura and mediastinum confirmed on subsequent biopsy. 

After subsequent surgical resection and initiation of adjuvant chemotherapy with cisplatin, doxorubicin, cyclophosphamide and dexamethasone, the LAEs normalized (AST 17 IU/L, ALT 12 IU/L, total bilirubin 0.2 mg/dL). Work-up for concomitant myasthenia gravis was negative.

**Figure 3:**
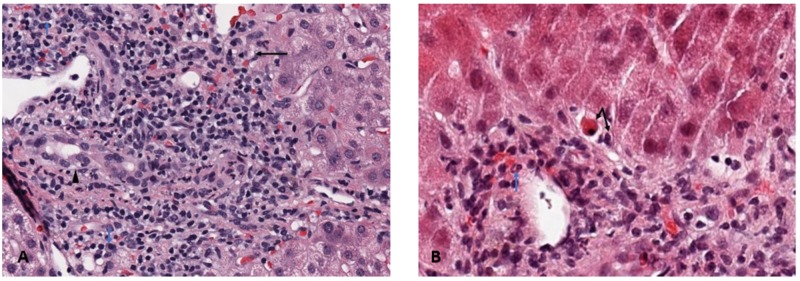
Follow-up Liver Biopsy - 3A: Predominant lymphocytic interface hepatitis (black arrows) with occasional plasma cells (blue arrow) and bile duct injury. No bile plugs. 3B: Interface hepatitis with an acidophil body (piecemeal necrosis)

Five months after the completion of chemotherapy and discontinuation of corticosteroids, his LAEs began rising, now with AST 240 IU/L, ALT 373 IU/L, and total bilirubin 5.5mg/dL. The patient was asymptomatic with exception of mild fatigue. He denied any further use of work-out supplements. A second liver biopsy was performed which demonstrated predominantly lymphocytic interface hepatitis with occasional plasma cells without cholestasis as previously seen. These findings are pathognomonic features of autoimmune hepatitis (Figure 3A, 3B).

## Discussion

Thymomas are rare neoplasms of the thymus, a primary lymphoid organ whose main function is T-cell maturation and differentiation. During the T-cell maturation process in the thymus, T-cells undergo both positive and negative selection. Positive selection ensures functionality of major histocompatibility complexes (MHC). Negative selection ensures T-cell non-reactivity to self-antigens, thereby preventing development of autoimmunity. Thymomas are commonly associated with immune-mediated paraneoplastic syndromes due to the neoplasm’s unchecked thymopoietic activity [2].

The tumor cells continue to allow T-lymphocyte differentiation without undergoing negative selection, leading to humoral and/or cellular loss of tolerance to self-antigens and subsequent cross-reactivity with other self-antigens in the periphery [1]. There are three main theories that attempt to explain the pathophysiology of thymomas and autoimmunity. The first is the “escape" theory, which hypothesizes that immature T-cells “escape” the neoplastic thymic environment before undergoing negative selection in the medulla due to disorganization caused by the neoplastic process. The second theory, the “genetic” theory, hypothesizes that dysplastic genetic defects from rapid proliferation of neoplastic cells lead to impairment of the negative selection process. The third theory is more specific, citing a mutation in the AIRE gene, which is directly involved in the negative selection process [3].

Once released by the neoplastic thymus, these auto-reactive T-lymphocytes can persist for months to years in the periphery, thereby explaining the delayed resolution or appearance of the paraneoplastic syndrome, even after resection of the thymoma, such as the persistently elevated liver-associated enzymes in our patient’s case [4]. Outside myasthenia gravis, thymectomy has not been shown to consistently lead to resolution of other paraneoplastic autoimmune disorders. This may suggest that T-cells lacking self-tolerance had already been exported to the peripheral immune system prior to thymectomy and continue to persist despite tumor resection [3].

Paraneoplastic syndromes are known to be associated with thymoma, most commonly myasthenia gravis, cited to affect 50% of all thymoma patients [5]. Other paraneoplastic syndromes induced by thymomas are not as well described in the literature. Upon literature review, autoimmune processes affecting the nervous system are most common, including neuromyotonia, psychosis and polymyositis. Outside the nervous system, paraneoplastic syndromes described include autoimmune thyroid diseases (both Grave's disease and Hashimoto’s thyroiditis), hematologic aplasias, cutaneous syndromes (pemphigous, vitiligo), systemic lupus erythematous and glomerulonephritis [6]. On our review of the medical literature, we found fewer then 10 reports of autoimmune hepatitis secondary to a thymoma and were only able to verify 4 of those reports. In a 2015 study of 85 patients with thymoma, 55% presented with autoimmune disease, only one of which was autoimmune hepatitis. In their subsequent review of past literature, 9 cases were cited. Of these nine cases, the diagnosis of autoimmune hepatitis was made simultaneously with the diagnosis of thymoma in seven of them. One of the other cases developed autoimmune hepatitis a few months after thymoma diagnosis and the other developed AIH four years after thymoma diagnosis. Most of the patients were young, with a mean age of 32 years old. Five of the nine were of Asian descent, suggesting a possible genetic pre-disposition. Seven of the nine patients had other autoimmune disorders in addition to autoimmune hepatitis, including polymyositis (n=2), Hashimoto’s thyroiditis (n=2), diabetes mellitus (n=1), and an unspecified connective tissue disorder (n=1). Six of the nine patients responded well to immunosuppressive therapy, similarly to the patient we present here [3]. 

This case report was presented as a poster at the national American College of Gastroenterology Annual Meeting in Philadelphia, PA on October 7, 2018 (https://eventscribe.com/2018/ACG/ajaxcalls/PosterInfo.asp?PosterID=160403&efp=RFNSWFFHSFY2NDI0&rnd=0.3753992).

## Conclusions

Thymomas are rare neoplasms of the thymus and are often associated with immune-mediated paraneoplastic syndromes, most commonly, myasthenia gravis. Other less common paraneoplastic syndromes are possible and should be evaluated based on individual patient presentation. There is still much to be learned about the mechanism of autoimmunity by thymomas. The paraneoplastic syndrome often coincides with thymoma diagnosis, but is also known to precede thymoma discovery and in some cases, appear much later in the disease course due to active metastases and activated T-lymphocytes that persist in the periphery. Autoimmune hepatitis should be considered as a potential paraneoplastic syndrome in patients with thymoma and elevated liver associated enzymes. Additionally, in the setting of nutritional supplement use, the diagnosis of autoimmune hepatitis can be easily missed due to anchoring bias towards drug-induced liver injury, a much more common diagnosis with similar appearance on histology. Physicians should continue to report paraneoplastic syndromes induced by thymomas in order to gain a more accurate perception of incidence. 
